# Obesity prevention and the Health promoting Schools framework: essential components and barriers to success

**DOI:** 10.1186/s12966-015-0167-7

**Published:** 2015-02-13

**Authors:** Rebecca Langford, Christopher Bonell, Hayley Jones, Rona Campbell

**Affiliations:** DECIPHer, School of Social and Community Medicine, University of Bristol, Canynge Hall, 39 Whatley Rd, Bristol, BS8 2PS UK; Social Science Research Unit, Institute of Education, University College London, 20 Bedford Way, London, WC1H 0AL UK; School of Social and Community Medicine, University of Bristol, Canynge Hall, 39 Whatley Rd, Bristol, BS8 2PS UK

**Keywords:** Children, Adolescents, Interventions, Schools, Physical activity, Healthy eating, Process evaluation, Health promoting Schools

## Abstract

**Background:**

Obesity is an important public health issue. Finding ways to increase physical activity and improve nutrition, particularly in children, is a clear priority. Our Cochrane review of the World Health Organization’s Health Promoting Schools (HPS) framework found this approach improved students’ physical activity and fitness, and increased fruit and vegetable intake. However, there was considerable heterogeneity in reported impacts. This paper synthesises process evaluation data from these studies to identify factors that might explain this variability.

**Methods:**

We searched 20 health, education and social-science databases, and trials registries and relevant websites in 2011 and 2013. No language or date restrictions were applied. We included cluster randomised controlled trials. Participants were school students aged 4-18 years. Studies were included if they: took an HPS approach (targeting curriculum, environment and family/community); focused on physical activity and/or nutrition; and presented process evaluation data. A framework approach was used to facilitate thematic analysis and synthesis of process data.

**Results:**

Twenty-six studies met the inclusion criteria. Most were conducted in America or Europe, with children aged 12 years or younger.

Although interventions were acceptable to students and teachers, fidelity varied considerably across trials. Involving families, while an intrinsic element of the HPS approach, was viewed as highly challenging. Several themes emerged regarding which elements of interventions were critical for success: tailoring programmes to individual schools’ needs; aligning interventions with schools’ core aims; working with teachers to develop programmes; and providing on-going training and support. An emphasis on academic subjects and lack of institutional support were barriers to implementation.

**Conclusions:**

Stronger alliances between health and education appear essential to intervention success. Researchers must work with schools to develop and implement interventions, and to evaluate their impact on both health *and* educational outcomes as this may be a key determinant of scalability. If family engagement is attempted, better ways to achieve this must be developed and evaluated. Further evaluations of interventions to promote physical activity and nutrition during adolescence are needed. Finally, process evaluations must move beyond simple measures of acceptability/fidelity to include detailed contextual information to illuminate exactly what works, for whom, in what contexts and why.

## Introduction

Obesity is a pressing public health issue. In the past three decades rates of overweight and obesity have increased dramatically in most industrialised countries, with increases also observed in several low-income contexts [1]. This global epidemic is of particular concern for children and young people. Almost a third of children in America and a fifth in Europe are overweight or obese [2,3]. Childhood obesity strongly ‘tracks’ into adulthood [4], with implications for morbidity and premature mortality [5].

Obesity is a complex condition requiring equally complex solutions. The World Health Organization (WHO) suggests this requires action in multiple settings, using a variety of approaches and involving diverse stakeholders [6]:p16. A key element is complex, multi-component interventions implemented in schools targeting key determinants of obesity: namely, physical activity and nutrition [7].

One such approach is the WHO’s Health Promoting Schools (HPS) framework. The HPS framework recognises the inherent, reciprocal link between health and education: healthy children achieve better educational outcomes which, in turn, are associated with better health later in life [8]. Inspired by the principles of the Ottawa charter and cognisant of the failure of health education alone to improve health outcomes, the HPS framework takes an eco-holistic approach to creating school environments conducive to health and healthy behaviours [9].

While definitions vary [8-14], HPS initiatives comprise: (1) health education promoted through the formal school curriculum; (2) changes to the school’s physical and/or social environment; and (3) engagement with families and the wider community in recognition of the influence of these on children’s health.

The HPS framework has proved popular in tackling obesity and other important public health issues such as cardio-vascular disease and Type II diabetes [11,15-17]. Half of the 67 trials included in our recent Cochrane review of the HPS framework targeted physical activity and/or nutrition [17]. Overall, we found intervention effects for improvements in students’ levels of physical activity, physical fitness, and fruit and vegetable intake. We found no overall effect for reducing students’ fat intake. The evidence for BMI and zBMI (standardised by age and gender) was equivocal. Surprisingly, given the underlying aim of the HPS framework, no study presented data on student academic attainment or attendance [17].

Within these studies we identified considerable heterogeneity in intervention effects. Given the complexity of these interventions and the variability between studies, it is important to consider why some were effective while others were not. Process evaluations can suggest explanations, helping to identify what works, for whom, in what contexts and why [18].

The aim of this paper was to synthesise process evaluation data presented in these studies to identify factors that helped or hindered implementation and/or success. Our findings have implications for the development of future trials and the implementation of programmes beyond the trial context.

## Methods

### Inclusion criteria

Full details of the methods can be found in the Cochrane review [17]. We included cluster randomised controlled trials (RCTs), with clusters at the level of school, district or other geographical area. Participants were students aged four to 18 years attending schools/colleges. As the HPS framework is not necessarily a term recognised in all countries, we did not require interventions to be explicitly based on the HPS framework. Rather, to be eligible interventions had to demonstrate active engagement in all three HPS domains, namely: curriculum, environment, and families and/or communities. Control schools offered no intervention or standard practice, or implemented an alternative intervention that included only one or two of the HPS criteria. For the purposes of this synthesis of process data, studies were included if they: took an HPS approach; focused on physical activity and/or nutrition; and presented process evaluation data.

### Search strategy

We searched the following databases and trials registries using broad and inclusive search terms: ASSIA, Australian Education Index, British Education Index, BiblioMap, CAB Abstracts, Campbell Library, CENTRAL, CINAHL, Database of Educational Research, EMBASE, Education Resources Information Centre, Global Health Database, International Bibliography of Social Sciences, Index to Theses in Great Britain and Ireland, MEDLINE, PsycINFO, System for Information on Grey Literature in Europe, Social Science Citation Index, Sociological Abstracts, TRoPHI, Clinicaltrials.gov, Current Controlled Trials, and International Clinical Trials Registry Platform. We also searched relevant websites and reference lists of relevant articles. Searches were conducted in 2011 and 2013. No date or language restrictions were applied. One author performed an initial title screen, with a second screening a randomly-selected 10% of these for quality assurance (kappa score = 0.88). Thereafter, two reviewers independently screened abstracts and full texts to determine eligibility.

### Data extraction

For each study, two reviewers independently extracted data pertaining to: study location, target age group, study duration, intervention content, outcome data and basic process data.

We undertook a thematic synthesis, adapting existing methods described by Thomas and Harden [19] to identify themes relating to programme implementation. More detailed descriptions of methods and quantitative and qualitative process findings were extracted verbatim from each study report by RL, that is extracting exactly the same words as the study author(s)’ used. Data were also extracted from discussion sections of reports when this addressed implementation or reasons for intervention success or failure. These extracts were read and re-read, an initial set of codes being developed and applied to the data. Some codes were identified a priori, focusing on aspects of process (acceptability, fidelity) while others arose inductively from the data (family involvement, barriers/facilitators). A Framework [20,21] approach was used to manage the data and assist analysis, whereby data from each study were summarised within a matrix under the following themes: intervention acceptability; implementation fidelity; family involvement; barriers to implementation; facilitators of implementation. This method allowed identification of similarities and differences between studies within themes.

## Results

**Figure 1:**
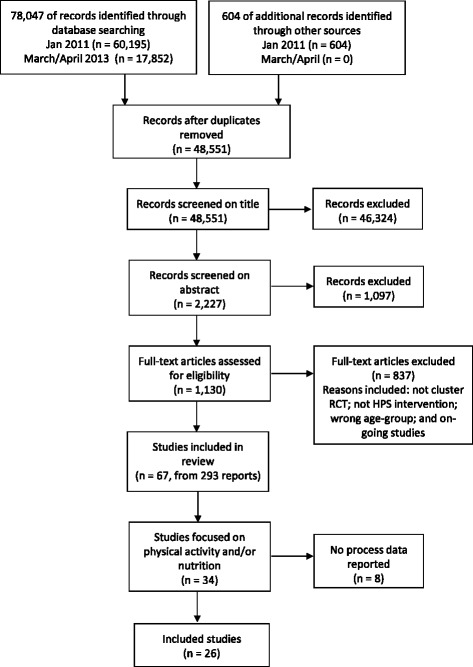
**Flow chart of study selection process.**

Our searches yielded 48,551 records (after deduplication), from which we identified 67 eligible studies (Figure 1). Of these, 34 focused on physical activity and/or nutrition. Twenty-six reported some process data and are the focus of this paper. Four studies focused solely on promoting physical activity, 11 on improving nutrition and 11 on physical activity and nutrition. Key characteristics of the interventions, including intervention activities carried out under the three HPS domains are presented in Table 1.

**Table 1:** **Characteristics of studies**

**Authors and programme name**	**Characteristics**	**HPS intervention elements**	**Summary of results**	**Process evaluation methods**
**Nutrition interventions**				
Bere et al. 2006 [22]	**Country:**	**Curriculum:**	No difference between intervention and control groups for fruit and vegetable intake.	Surveys to teachers, parents and students to assess participation and acceptability (assessed on likert scale).
*Fruits and Vegetables Make the Mark*	Norway	Curriculum was delivered in Home Economics lesson over a period of 7 months. Activities included preparing fruit/veg-based meals and snacks, taste testing, and monitoring of fruit and vegetable consumption over 3 days.		
	**Target group:**	**Environment**:		
	11-12 years	Schools encouraged to participate in the national fruit and vegetable subscription programme.		
	**Duration:**	**Family/community:**		
	6 months	Newsletters, parents meeting.		
Evans et al. 2013 [23]	**Country:**	**Curriculum:**	No difference between intervention and control groups for intake or portions of fruit and vegetable intake.	Questionnaires to teachers, parents and students to assess implementation, participation and acceptability (assessed on likert scale).
*Project Tomato*	UK	Teachers were provided with twelve lesson plans.		
	**Target group:**	**Environment:**		
	7-8 years	School health committee to co-ordinate activities.		
	**Duration:**	**Family/community:**		
	10 months	Advice, newsletters and take-home activity bags.		
Foster et al. 2008 [24]	**Country:**	**Curriculum:**	Incidence of overweight was significantly lower for intervention than control students (7.5% vs. 14.9%, adjusted odds ratio: 0.67, 95% CI 0.47 to 0.96).	Very minimal data provided on staff training and hours of nutrition education. No details on methods provided.
*School Nutrition Policy Initiative*	USA	50 hours of food and nutrition education provided per year. The curriculum was integrated into various classroom subjects.		
	**Target group:**	**Environment:**		
	9-12 years	Nutrition Advisory Group set up. Changes made to food sold in schools to ensure they met nutritional standards. Other activities included: limiting use of food as a reward, promoting active recess and providing healthy breakfasts.		
	**Duration:**	**Family/community:**		
	2 years	Report card nights, parent education meetings, and weekly nutrition workshops.		
Hoffman et al. 2010 [25]	**Country:**	**Curriculum:**	By end of year 2, intervention group consumed more fruit (but not vegetables) than the control group (34g vs. 23g, p<0.001).	Questionnaires to teachers, lunch aides and students to assess acceptability using 6-point likert scale. Unannounced fidelity checks and observations of lunchtime components. Log book of public service announcements kept.
*Athletes in Service, Fruit and Vegetable Promotion Program*	USA	The classroom component included the 5-A-Day Adventures CD-ROM.		
	**Target group:**	**Environment:**		
	5-7 years	Loudspeaker announcements and posters to promote fruit/veg. Lunch aides praised children eating fruit and vegetables and offered stickers.		
	**Duration:**	**Family/community**		
	2.5 years	Family homework assignments. Parents involved in creating a school cookbook.		
Hoppu et al. 2010 [26]	**Country:**	**Curriculum:**	No difference between intervention and control group for fruit/veg consumption.	Teachers, catering staff and students asked to give their opinions on the intervention (no further details provided on methods or questions asked). Teachers reported use of intervention materials using on-line system.
(no name)	Finland	Nutrition education was implemented by teachers during regular lessons.		
	**Target group:**	**Environment:**		
	13-14 years	Sugary snacks restricted and healthy alternatives encouraged. Drama workshops held.		
	**Duration:**	**Family/community:**		
	8 months	Parent information meeting. Healthy eating magazine.		
Lytle et al. 2004 [27]	**Country:**	**Curriculum:**	No significant differences between intervention and control groups for fruit and vegetable intake.	Lesson checklists and observations of classroom sessions. Teacher and student evaluations of curriculum acceptability assed via likert scale. Documentation of family participation via number of behavioural coupons returned and family homework assignments completed. Visits to food service teams. Logs of school nutrition advisory councils.
*TEENS*	USA	Ten nutrition education lessons were implemented in both grade 7 and 8. These sessions involved self-monitoring, goal setting, hands-on snack preparation, and skill development.		
	**Target group:**	**Environment:**		
	12-14 years	Changes made to school food service to improve nutritional quality of food. School Nutrition Advisory Councils created.		
	**Duration:**	**Family/community:**		
	2 years	Newsletters and behavioural coupons.		
Nicklas et al. 1998 [28,29]	**Country:**	**Curriculum:**	No significant differences between intervention and control groups for fruit and vegetable intake.	Observations and evaluation forms for training workshops. Logbooks document family involvement via newsletters/calendar distribution and attendance at parent-teacher meetings. Menu documentation, salad bar assessments and food use surveys. Monthly rates of participation in school lunches recorded.
*Gimme 5*	USA	Five (55 minute) themed workshops provided students with learning opportunities to develop knowledge, positive attitudes and skills necessary to increase fruit and vegetable consumption.		
	**Target group:**	**Environment:**		
	14-15 years	School-wide media marketing campaign was implemented including taste testing, posters, public service announcements and student contests. School meals modified to increase fruit and veg provision. Staff training.		
	**Duration:**	**Family/community:**		
	3 years	Parents’ brochures, newsletters and a seasonal food calendar. Events held at Parent-Teacher Organization meetings.		
Perry et al. 1998 [30,31]	**Country:**	**Curriculum:**	Intervention students consumed more servings of fruit (per 100 kcals, difference 0.41, P=0.02) and combined fruit and vegetable servings (per 100 kcals, difference 0.36, P=0.02) and less fat (as % of total kcal, difference −1.81, P=0.02).	Training participation rates and feedback from participants. Observations of classroom activities and lunchtimes.
*5 A DAY Power Plus*	USA	Sixteen 40–45 minutes classroom sessions were implemented twice a week for 8 weeks. Sessions included skills-building, problem-solving and taste-testing.		
	**Target group:**	**Environment:**		
	9-11 years	Changes made to school food provision. Catering staff training. Students were rewarded for eating fruits and vegetables during lunch.		
	**Duration:**	**Family/community:**		
	6 months	Home information/activity packs were sent home.		
Radcliffe et al. 2005 [32]	**Country:**	**Curriculum:**	No significant difference between intervention and control groups for % students skipping breakfast.	No details on methods but table provided number of schools implementing each intervention component.
(no name)	Australia	Variety of changes to the curriculum including: classes focusing on health, nutrition and breakfast; a unit on body image and healthy eating; breakfast information provided to teachers; development of breakfast recipe books and trailing of recipes etc.		
	**Target group:**	**Environment:**		
	12-13 years	Working group developed action plans. Variety of activities including: events to promote breakfast; designating a breakfast eating area; change to timetable to enable earlier morning snack times; trialling breakfast tuck shops; improving nutritional quality of breakfast foods sold at the tuckshop.		
	**Duration:**	**Family/community:**		
	11 months	Variety of activities including: newsletter; parent education forums; involving parents in classroom activities and special events etc.		
Reynolds et al. 2000 [33,34]	**Country:**	**Curriculum:**	Intervention children consumed significantly more fruit and vegetables than the control group (3.96 servings vs. 2.28, P<0.0001).	Questionnaire to curriculum co-ordinators to assess acceptability (on 5-point likert scale). Classroom observations and checklists. Cafeteria observations. Interviews with food service managers. Assessments of family involvement through checklists, assessments of homework completion and telephone parent surveys.
*High 5*	USA	Nutrition curriculum (14 lessons) d included modelling, self-monitoring, problem-solving, reinforcement, taste testing and other methods.		
	**Target group:**	**Environment:**		
	9-10 years	Food service managers training. Cafeteria was rated on a monthly basis and given 2, 3 or 4stars based on their completion of 10 intervention activities.		
	**Duration:**	**Family/community:**		
	1 year	Parents’ ‘kick-off’ night. Family homework assignments.		
Te Velde et al. 2008 [35,36]	**Country:**	**Curriculum:**	At first follow-up intervention children reported greater intake of fruit and vegetables compared to controls. However, by second follow-up (one year later) a significant effect was only seen in one country (Norway, 91.5 additional grams per day, 95% CI 49.8 to 133.2, p=0.04).	Teacher questionnaire used to assess fidelity (composite score created ranging from 0–16). Student survey to assess acceptability on 3-point likert scale for different intervention elements. Assessments of family involvement via completion of family homework assignments, use of intervention’s computer programme, and receipt of newsletters.
*Pro Children Study*	Netherlands, Norway, Spain	16 worksheets aimed at increasing knowledge, awareness and skills. Included taste testing activities and computerised tailored feedback.		
	**Target group:**	**Environment:**		
	10-12 years	Free fruit/veg provided, Changes to school food provision to increase amount of fruit/veg available.		
	**Duration:**	**Family/community:**		
	2 years	Family homework assignments, newsletters and a parent version of the web-based computer-tailored tool.		
**Physical activity interventions**				
Eather et al. 2013 [37]	**Country:**	Home activity programme comprised of 20 minutes physical activity, three times a week for 8 weeks. Work booklets, information and fitness challenges sent to parents.	Significant improvements in intervention group found for: fitness (adjusted mean difference, 1.14 levels, p < 0.001), BMI (mean, − 0.96 kg/m2, p < 0.001) zBMI z-score mean − 0.47 z-scores, p < 0.001), flexibility (sit and reach mean, 1.52 cm, p = 0.0013), muscular fitness (sit-ups) (mean 0.62 stages, p = 0.003) and physical activity (mean, 3253 steps/day, p < 0.001).	Questionnaires to teachers and students to assess participation and satisfaction on six-point likert scale.
*Fit-4-Fun*	Australia			
	**Target group:**			
	10-12 years			
	**Duration:**			
	8 weeks			
Kriemler et al. 2010 [38]	**Country:**	**Curriculum:**	Intervention children showed improvements in skinfold thickness (−0.12, 95% CI −0.21 to −0.03), fitness (0.17, 95% CI 0.01 to 0.32), school MVPA (1.19, 95% CI 0.78 to 1.6) and all-day MVPA (0.44, 95% CI 0.05 to 0.82) and total physical activity in school (0.92, 95% CI 0.35 to 1.5).	Questionnaires to teachers and students to assess acceptability on 6-point likert scale.
*KISS*	Switzerland	Two additional PE lessons a week were implemented by specialist PE teachers.		
	**Target group:**	**Environment:**		
	6-7, 10–11 years	Several short activity breaks (2–5 minutes) were introduced during academic lesson every day.		
	**Duration:**	**Family/community:**		
	11 months	Flyers on health topics were sent to parents		
Simon et al. 2006 [39]	**Country:**	**Curriculum:**	Intervention students had a lower increase in BMI (p<0.01) and age/gender-adjusted BMI (p<0.02). They also had increased participation in supervised physical activity (p<0.001), a decrease in TV/video viewing (p<0.01) and an increase in high-density cholesterol concentrations (p<0.001).	Quantitative documentation of number of activities provided and individual attendance at sessions. Number of school hours devoted to curriculum and school debates. Actions initiated by or in collaboration with outside partners were recorded.
*ICAPS*	France	Curriculum focused on physical activity and sedentary behaviours. Aimed to transmit knowledge and skills about physical activity.		
	**Target group:**	**Environment:**		
	11-12 years	Increased opportunities for physical activity offered at breaks, at lunchtimes and after school.		
	**Duration:**	**Family/community:**		
	4 years	Parents and teacher meetings. Policy makers of local communities were requested to provide a supportive environment that promote physical activity. For example, free/low-cost entry to sports facilities.		
Wen et al. 2008 [40,41]	**Country:**	**Curriculum:**	No difference between intervention and control students reporting walking to school.	Semi-structured qualitative interviews with principals and/or teaching co-ordinators to assess fidelity and acceptability.
(no name)	Australia	Home to school mapping exercise’ used to help students plan their active journey to high school next year. Some schools also used pedometers and an associated classroom program.		
	**Target group:**	**Environment:**		
	9-11 years	A consultation group comprised of teachers, parents and officers from local councils set up to encourage active commuting. Banners provided for schools. Walk Safely to School Day activities held.		
	**Duration:**	**Family/community:**		
	2 years	Information on active travel provided to parent. Parent events and walks. Newsletters. Local councils reviewed safety and walkability of nearby participating schools and worked to make improvements.		
**Physical Activity + Nutrition Interventions**				
Brandstetter et al. 2012 [42]	**Country:**	**Curriculum:**	No effect found on BMI, waist circumference and skin-fold thickness after adjustment for time lag between baseline and follow-up.	Questionnaire to teachers to assess fidelity (teachers asked to indicate which teaching units has been used in class).
*URMEL ICE*	Germany	29 units (each 30–60 minutes) implemented over one school year. Focused on reducing the amount of sugary drinks consumed and screen time, and increasing physical activity.		
	**Target group:**	**Environment:**		
	7-8 years	Two short blocks of physical activity exercises (each 5–7 minutes) were implemented every day. Teachers training.		
	**Duration:**	**Family/community:**		
	9 months	Family homework assignments and training and information materials.		
Caballero et al. 2003 [43-45]	**Country:**	**Curriculum:**	No difference between intervention and control for BMI or other anthropometric measures or physical activity levels. Intervention students reported lower total daily energy intake (1892 vs. 2157 kcal/d, p=0.003) and percentage of energy from fat (31.1% vs. 33.6%, p=0.001) than control students.	Quantitative assessments of the four main components of the intervention, including attendance log for training sessions and family events, PE calendars, kitchen visits and parent/student evaluation forms.
*Pathways*	USA	Classroom curriculum designed to physical activity and nutrition. In 3rd and 4th grades, two 45-minute lessons delivered for 12 weeks. In 5th grade this decreased to 8 weeks.		
	**Target group:**	**Environment:**		
	8**–**9 years	School food service guidelines issues to decrease fat content of meals. Minimum of three 30-minute sessions of MVPA per week. Exercise breaks to promote physical activity in the classroom. Teacher training.		
	**Duration:**	**Family/community:**		
	3 years	Family action packs. Family events, included cooking demonstrations.		
Crespo et al. 2012 [46]	**Country:**	**Curriculum:**	No difference between intervention and control groups for BMI.	Direct observations and audit tools used to assess fidelity of intervention components (no further details provided).
*Aventuras para Niños*	USA	SPARK physical activity curriculum implemented.		
	**Target group:**	**Environment:**		
	5-8 years	Improvements were made to school playgrounds and salad bars. Physical activity equipment provided. Posters. Student newsletters.		
	**Duration:**	**Family/community:**		
	5 semesters	Improvements made to community parks. Local restaurants asked to create healthy children’s menus. Frequent produce buyers cards distributed.		
Foster et al. 2010 [47,48]	**Country:**	**Curriculum:**	No difference between intervention and control groups for combined prevalence of overweight/obesity. However, intervention students had greater reductions in zBMI, waist circumference above 90th percentile, fasting insulin levels and prevalence of obesity (p<0.04 for all).	Structured observations of PE lessons, curriculum components, school cafeterias and media campaigns. Qualitative interviews with key staff (e.g. physical activity co-ordinators).
*HEALTHY*	USA	A classroom based curriculum (FLASH - Fun Learning Activities for Student Health) targeting self-awareness, knowledge, behavioural skills and peer involvement for behavioural change.		
	**Target group:**	**Environment:**		
	11-14 years	Changes made to school meals to improve nutritional quality. Changes made to PE lessons to increase the amount of time spent in MVPA.		
	**Duration:**	**Family/community:**		
	3 years	Family outreach newsletters and take-home packs.		
Grydeland et al. 2013 [49]	**Country:**	**Curriculum:**	No overall effect found for impact on BMI, but positive intervention effects were found for BMI (p=0.02) and zBMI girls (p=0.003) but not boys.	Unpublished process data mentioned in discussion of paper. Log books and questionnaires with teachers, parents and children.
*Health in Adolescents (HEIA)*	Norway	Five class-room sessions on nutrition and physical activity were delivered by teachers to students during the 6th grade.		
	**Target group:**	**Environment:**		
	11-12 years	Short (10 minute) physical activity breaks and fruit/veg breaks held once a week during lessons. Sports equipment was provided. Active commuting campaigners. Pedometers given out. PE teacher training.		
	**Duration:**	**Family/community:**		
	20 months	Parent fact sheets. Family homework assignments.		
Haerens et al. 2006 [50]	**Country:**	**Curriculum:**	Overall, no significant effect on BMI was found, although a positive effect was seen for girls (p<0.05).	Teacher questionnaire to assess level of implementation assessed on a 5-point scale.
(no name)	Belgium	Computer-tailored intervention to promote physical activity and healthy eating with personalised feedback.		
	**Target group:**	**Environment:**		
	12-14 years	Extra opportunities to be physically activities during breaks, at lunchtime and after school. School health workgroups.		
	**Duration:**	**Family/community:**		
	2 years	Parent meetings, information leaflets and CD-rom provides.		
Luepker et al. 1996 [51]	**Country:**	**Curriculum:**	Intervention students reported more daily vigorous activity than controls (59 vs. 47 minutes, p<0.003) and greater reductions in daily energy intake from fat (2.4% vs. 0.4% reductions, p<0.001).	Questionnaires to assess acceptability, attendance and feedback from training sessions, checklists and documentation logs for intervention activities, structured observations of intervention activities.
*CATCH*	USA	Classroom curricula implemented in grades 3–5 for between 5 and 12 weeks (depending on grade). Each lesson was 30–40 minutes. The curricula targeted psychosocial factors and skills development.		
	**Target group:**	**Environment:**		
	8-9 years	Changes made to school meals to improve nutritional content. Catering staff training. Changes to PE lessons to increase time spent in MVPA. Teacher training.		
	**Duration:**	**Family/community:**		
	3 years	Activity packs were sent home to be completed by students and parents together. Family fun nights.		
Sahota et al. 2001 [52,53]	**Country:**	**Curriculum:**	No difference between intervention and control group for overweight/obesity. Intervention children consumed more vegetables than control group (weighted mean difference: 0.3, 95%CI 0.2 to 0.4).	Teacher surveys (no further details provided). Meals monitored by collection of monthly menus, observations and discussions with staff.
*APPLES*	UK	Nutrition education incorporated into the curriculum, healthy eating lessons delivered by the project dietician and ‘Fit is Fun’ programme incorporated into physical education lessons.		
	**Target group:**	**Environment:**		
	9-11 years	Teacher training, modification of school meals and the development of school action plans designed to promote healthy eating and physical activity.		
	**Duration:**	**Family/community:**		
	10 months	Consultation with parents about what the intervention should include. Parents were invited to help run sessions. Information on intervention sent out to parents.		
Sallis et al. 2003 [54]	**Country:**	**Curriculum:**	Total physical activity levels improved in the intervention group compared to controls (d=0.93, p<0.009). Subgroup analyses revealed the intervention to only be effective for boys. BMI was reduced in boys in the intervention group (d-0.83, P=0.04). No effect seen for total fat or saturated fat.	Discussion section of paper describes problems encountered but no detail provided on how these data were collected.
*M-SPAN*	USA	Changes to PE lesson context, structure and teacher behaviour to increase physical activity.		
	**Target group:**	**Environment:**		
	11-14 years	Physical activity was promoted throughout the school day (e.g. during breaks and lunchtimes). School policies to support physical activity and healthy eating implemented. Changes made to the nutritional quality of food offered in schools. Student health committees set up to implement monthly health-related activities.		
	**Duration:**	**Family/community:**		
	2 years	Intervention was promoted to parents via articles in the school newsletter, posters and brochures at open houses and presentations to Parent Teacher Association meetings.		
Trevino et al. 2004 [55,56]	**Country:**	**Curriculum:**	Fitness scores (p=0.04) and dietary fibre intake (p=0.09) increased significantly in intervention children compared to controls.	Focus groups conducted to explore barriers to family involvement. No details provided for methods for assessing fidelity/intensity.
*Bienestar*	USA	50 × 45 minute health education sessions throughout the intervention. Curriculum focuses on nutrition, physical activity, self-esteem, self-control, and diabetes mellitus.		
	**Target group:**	**Environment:**		
	9-10 years	School food service staff receive nutritional training. *Bienestar* health club held once a week after school.		
	**Duration:**	**Family/community:**		
	5 months	Variety of parent ‘fun’ activities are held including: cooking demonstrations, salsa classes and games. Parent meetings.		
Williamson et al. 2012	**Country:**	**Curriculum:**	No differences between intervention and control students for body fat and BMI z-scores.	Questionnaires and observations used to assess integrity of delivery (No further details provided). Tracking system used to monitor usage of internet component
*Louisiana (LA) HEALTH* [57,58]	USA	Weekly classroom lessons (20–25 mins) on healthy eating and exercise implemented by teachers, as well as additional internet lessons.		
	**Target group:**	**Environment:**		
	9-12 years	Health promotion campaigns carried out in classrooms, hallways and other locations within the school. Modifications to school food provision to increase healthy options. Catering staff training. Vending machines provide healthy options. Regular 5 minute activity breaks in classrooms. Physical activity equipment.		
	**Duration:**	**Family/community:**		
	2.5 years	Bi-monthly newsletters sent home to parents. Family homework assignments. Healthy menus sent to parents.		

*Abbreviations* used in table: *BMI* (Body Mass Index), *zBMI* (Body Mass Index, standardized by age and gender), *MVPA* (moderate-to-vigorous physical activity), *PE* (Physical Education), *CI* (confidence interval).

### Countries

Thirteen studies were conducted in the USA [24,25,27,28,30,33,43,46,47,51,54,55,58]. A further 10 were implemented in Europe; two in the UK [23,52], two in Norway [22,49], one multi-country study (involving The Netherlands, Spain and Norway) [35] and one study each in Belgium [50], Finland [26], France [39], Germany [42] and Switzerland [38]. Three studies were conducted in Australia [32,37,40].

### Age groups

Of the 26 included studies, there were almost three times as many interventions conducted with younger children (≤12 years) compared to older children (19 vs. 7 studies, respectively). Among the former, most targeted students aged between 8-12 years (11 studies), while five included younger children. Studies focusing on older students tended to be conducted with 12-14 year olds. Only one study was implemented in grade 9 (14-15 years).

### Quality of process data

The methods used to collect process data are summarised in Table 1. The quality and extent of process data varied greatly. Some studies conducted extensive process evaluations, examining different elements of the intervention implementation. Most studies only focused on fidelity and acceptability using quantitative data, with diverse methods and scales bespoke for each intervention (e.g. questionnaires, log-books, structured observations). No study reported on the reliability or validity of these scales. Other studies provided extremely limited process data; it was often unclear how these were collected. Authors’ conclusions about facilitators and barriers to implementation were included in some discussion sections but the evidence for these was generally unclear. However, these insights appeared to us useful and are included in our analysis. Few studies provided qualitative data. We summarise key themes below.

### Key themes

#### Acceptability

Where reported, acceptability of the intervention to students, parents, and teaching or catering staff was generally high. Some studies provided quantitative assessments of acceptability. For example, teachers in the study by Bere et al. [22] all rated the intervention as ‘good’ or ‘very good’, while 70% of teachers and 90% of students participating in the *KISS* study reported they enjoyed the programme and wanted it to continue the following year [38]. Other studies reported acceptability in more general terms. For example, Hoppu et al. merely stated that ‘most of the feedback [on the intervention] was positive’ [26]:p975. Resources (such as sports equipment [48], stickers to promote fruit and vegetable intake [25] or pedometers [41]) were often highly rated by teachers and students. Training and support provided to teachers were also highly appreciated [44,52]. One study conducted structured interviews with Physical Education (PE) teachers to assess the intervention’s acceptability [48]. Some teachers were initially resistant to the new curriculum and implementation was lower in the early stages of the project. However, resistance lessened as they became familiar with the curriculum and could see the positive effect on student behaviour and activity levels.

#### Fidelity

Where reported, intervention fidelity varied. Some studies reported high levels of intervention fidelity [30,42,43,51,52]. In most studies, this was expressed as a percentage of intervention activities successfully implemented. For example, the *CATCH* trial reported 90% of food guidelines were met, 80% of PE activities were implemented and 88% of curriculum sessions were completed without modification [51]. Other studies reported much lower rates of implementation [22,23,27,34,46,54,58]. For example, one study [23] reported that despite high levels of acceptability, only 21% of intervention materials were implemented. Another [54] reported numerous problems with implementation, such as lack of volunteers or food preparation guidelines not being followed. Reynolds et al. [34] noted that taste-testing sessions were less likely to be undertaken because of the effort and disruption these caused. Importantly, they also noted that African-American and low socio-economic-status (SES) students were likely to receive lower doses of intervention activities, raising equity concerns. In the absence of additional contextual data, the authors were unable to explain this difference.

#### Family involvement

Despite being one of the three HPS domains, studies consistently identified engaging families as the most challenging and least successful intervention element. Almost all studies reporting on this indicated family engagement was low (typically only one-third to one-half of parents participating in *any* intervention activity) and authors frequently commented on the challenges of involving parents. The *ICAPS* trial noted parental attendance at meetings was poor (25-40%), particularly in low SES areas [39]. Similarly, Eather and colleagues acknowledged ‘parents are notoriously difficult to engage’ and many students were not supported at home in completing their intervention’s home-based activities [37]:p16. Two studies [36,41] identified language barriers in engaging non-English speaking parents. Even in the *CATCH* study where 70% of parents participated in some intervention activities, the authors noted the intensity of the family component was very low and thus unable to produce significant changes in children’s health behaviours [59].

A report on the *Bienestar* programme targeting Mexican Americans [55] identified very low family participation rates (17%), and study investigators conducted focus groups with parents to explore why [56]. Explanations included: misunderstanding the purpose of the programme and the family events offered; practical issues such as lack of transportation, babysitting or limited income; time constraints and conflicts with work schedules; embarrassment concerning parents’ own education and/or literacy levels; expectations of ‘boring’, didactic, teacher-led meetings; too many meetings offered; and assumptions that meetings were just for mothers and excluded other family members. In response, the program was modified to encourage greater participation leading to an increase in parental involvement, but nonetheless still only resulting in about a third of parents becoming involved (increase from 17% to 37%).

#### Facilitators of implementation

Making interventions relevant to the specific school context was noted as important. Needs assessments were undertaken in schools in some studies [32,52] helping to create interest and motivation within schools. Several studies encouraged schools to tailor intervention components to local needs. Equally, materials and messages needed to be culturally relevant, especially when targeting specific ethnic groups [43,55], helping create local ownership.

Good communication and thorough training were also noted as essential. Nicklas and O’Neill [29] explained that schools need to feel confident they are able to carry out the required tasks and adequate training is essential in achieving implementation fidelity. Researchers on the *5 A Day Power Plus* study similarly recognised training and staff development as critical to implementation [31]. Teachers in this study received on-going training with one-on-one feedback and support. To facilitate this, the study reimbursed schools for the cost of substitute teachers and paid catering staff to attend training.

Understanding schools’ ‘core business’ also appeared critical. As Nicklas and O’Neill pointed out, successful school health interventions’ objectives aligned with teachers’ goals for their students [29]. Radcliffe et al. [32], for example, explained that schools in their study saw the intervention programme as related to the core business of the school. A needs assessment identified lack of breakfast as a particular problem and teachers were convinced that this was associated with poorer concentration and classroom behaviour. Disappointingly this study did not go on to measure the intervention’s impact on these educational outcomes.

Working with schools to develop programmes was similarly important. Teachers could help develop both the intervention programme and its delivery plan to ensure relevancy and increase implementation fidelity [36]. Participation from students could also be useful [26]. Brandstetter et al. [42] advocated the need for pragmatic programmes that fit within the existing curriculum and school structures without creating additional demands on teachers.

#### Barriers to implementation

Competing priorities and lack of institutional support were noted as barriers to successful implementation. Both the *HEALTHY* [48] and *Pathways* [45] studies cited the emphasis on academic subjects over PE as hindering implementation. One programme co-ordinator from the *HEALTHY* study noted that ‘at times, the administration was pulling students out of PE to do some academic testing, and we had situations where students were working on writing assignments during PE because of pressures from administration’([48]:p313). Similarly, Story and colleagues suggested that implementation of the *5 A DAY Power Plus* programme in the fifth grade dropped because media reports of low academic test scores within the district meant teachers refocused only on ‘teaching the basics’ [31]. The intervention programme was thus an additional pressure which could easily be dropped. Other studies suggested that preparation for a forthcoming educational inspection or a general lack of time compromised teachers’ ability to engage in and deliver the intervention [27,36,45,52].

Numerous practical issues also presented challenges to implementation including: lack of space to deliver PE lessons [45]; difficulties in delivering hands-on taste testing sessions [34]; teacher absences or rapid staff turn-over [41,52]; high student-to-teacher ratios [48]; and lack of volunteers to run after-school physical activities [54]. Concerns over teacher burn-out and disruptive student behaviour were also mentioned [45,48].

Some issues that affected intervention delivery and success were beyond schools’ control. Fry and colleagues [41], for example, described how creating safe walking route to schools meant tackling local infrastructure, traffic management and access to public transport. Some school principals talked to local authorities about addressing these issues with varying success. Sallis et al. [54] identified the need for school food services to be financially self-supporting as the greatest barrier to improving student nutrition. Offering unfamiliar (and potentially unpopular) healthy foods posed too great a financial risk for catering services and thus disincentivised change. In addition, a centralized kitchen system meant schools had little control over ingredients or preparation.

Finally, although interventions must be of sufficient length and intensity to enable behavioural change and health impacts, optimal duration is not clear from these studies, there being no clear association between intervention duration and health impact.

## Discussion

The HPS framework is generally effective at increasing physical activity, fitness and fruit and vegetable intake in school students [17]. This paper looked in more detail at the implementation of physical activity and/or nutrition interventions and identified key factors helping or hindering implementation and/or success.

### Summary of main findings

Process evaluations revealed high levels of acceptability among teachers and students, but implementation fidelity varied considerably across trials. In particular, involving families, despite being a key part of the HPS approach, was reported as highly challenging. Essential elements of interventions included: tailoring programmes to individual schools’ needs; aligning interventions with schools’ core aims; working with teachers to develop programmes and increase ownership; and providing on-going training, support and communication. The emphasis on academic subjects (and the corresponding low value placed on health initiatives), and lack of institutional support were cited as barriers to implementation.

Many of these findings are congruent with conclusions from other studies examining effective elements of school-based interventions. A recent narrative synthesis of qualitative studies adopting the HPS approach [60] identified the importance of institutional support, assessment of school needs, ownership of programmes, adequate training and tailoring of intervention components to local contexts. This review also noted the low value placed on health versus academic achievement as a barrier to effective implementation. Similar findings regarding intervention development and implementation are also noted in Peters et al.’s recent review of school health promotion [61] and in guidelines for HPS produced by the International Union for Health Promotion and Education [14].

### Limitations

Most studies lacked detailed description of intervention components and activities which would enable replication in other contexts. Equally, the quality of process data varied considerably and was often poorly reported. While providing some useful insights, much of the process data presented by these studies was disappointing in terms of its scope and depth. Process data generally consisted of quantitative assessments of acceptability and/or fidelity. While important, these are insufficient to explain why some interventions failed while others succeeded. For example, while studies which reported positive intervention impacts also reported high levels of implementation fidelity, so did many other studies which found no such positive effects. The most useful insights into intervention success often came from authors’ reflections reported in the discussion sections of papers, despite the evidence for many such claims being unclear. However, it should be noted that findings reported here are often based on reports from just one or two studies.

Better designed, more comprehensive process evaluations that go beyond mere quantitative assessments of acceptability and/or fidelity are required. These should provide greater insight into the context in which interventions are implemented and how this can affect intervention success. It is also notable that almost a quarter of the 34 physical activity and/or nutrition interventions identified by the Cochrane review provided no process data and were thus excluded from this analysis.

The publication of the template for intervention description and replication (TIDieR) checklist and guide [62] and the recently published process evaluation guidance [63] from the UK’s Medical Research Council are welcome developments. The adoption of these guidelines by scientific journals may prove important in raising both the profile and quality of reporting of trials and associated process evaluations. This is particularly important for multi-component, complex interventions where it is important to identify, first, what is being standardised in the intervention (intervention components or steps in the change process [64]) and, second, what works, for whom, in what circumstances and why [18].

That this study focuses only on RCT evidence is both a strength and a limitation. While evidence from RCTs provides the most reliable means of assessing intervention effectiveness, we acknowledge that many evaluations of the HPS approach do not use this methodology and thus were excluded from this review [11,14,15]. However, findings reported here are congruent with reviews of the wider evidence base [14,16] and contribute to the emerging picture of how best to improve physical activity and nutrition in schools. Implementation trials (including process evaluations) that evaluate the roll-out of successful programmes would extend our understanding of how to implement such interventions in ‘real world’ settings.

### Implications for policy and research

Our findings raise three challenges for both policy makers and researchers. First, we need greater integration between health and education [65]. As suggested by the findings described above, schools are more likely to engage in health interventions if they fit with institutional priorities, namely improving educational attainment. It is disappointing that none of these HPS interventions measured outcomes such as academic test scores, attendance, attention, concentration, behaviour in the classroom or attitude towards school. Overweight and obesity have been found to be associated with poor academic performance [66]. There is also some evidence to suggest physical activity [67,68] and nutrition (particularly breakfast schemes) [68-71] can improve academic achievement. However, these data are often from methodologically weak studies and causality has yet to be demonstrated [72]. It is imperative that rigorous RCT evaluation studies include both health *and* educational outcomes to determine effectiveness, thus speaking to policy makers in health and education sectors alike.

Second, school health researchers need to carefully consider the importance of family involvement within HPS interventions. Evidence for the impact of family involvement in school-based obesity interventions remains inconclusive; some reviews suggest its importance [7,73] while others report no consistent pattern [74,75]. It may be that this aspect of the HPS framework is impractical in some or all schools and interventions would do better to focus resources on ‘in-school’ activities. Or it may simply be, as this review has found, that current approaches to parental involvement are inadequate (for example, newsletters or information evenings) and more innovative methods are required. Further research – both qualitative and quantitative – is needed to address these questions.

Finally, we need more evaluations (including process evaluations that go beyond quantitative measures of fidelity and acceptability) of interventions to promote physical activity and nutrition during adolescence. Physical activity levels are known to decline during teenage years, particularly in young women [76,77]. Adolescence also represents a period when young people start to make their own choices over the food they eat and how they spend their time [78]. The lack of research into this critical age period is therefore disappointing and represents a missed opportunity for public health impact.

## Conclusion

The HPS framework has been shown to be effective overall in improving physical activity and nutrition, the key determinants of overweight and obesity [17]. The process data reported in these trials offer important insights into essential elements of success, as well as the challenges to implementation which need to be addressed at the outset of any new programme. These data suggest that the success of the HPS approach lies in creating effective partnerships between researchers, schools and families.
